# MUC13 promotes the development of esophageal cancer by upregulating the expression of o-glycan process-related molecules

**DOI:** 10.1007/s12672-023-00713-3

**Published:** 2023-07-03

**Authors:** Yi Han, Gang Chen, Shiyu Liu, Guangqing Zhou, Xinxin Xu, Haihan Zhang, Zhentao Li, Chuannan Wu, Yulan Liu, Kai Fang, Guangxia Chen

**Affiliations:** 1 Department of Gastroenterology, The First People’s Hospital of Xuzhou, Xuzhou Municipal Hospital Affiliated to Xuzhou Medical University, Xuzhou, 221002 China; 2 Department of Plastic Surgery, The Affiliated Hospital of Nanjing University of Chinese Medicine, Nanjing, 210029 China; 3 Xuzhou Medical University, Xuzhou, 221002 China; 4 The Second Affiliated Hospital of Chengdu Medical College, China National Nuclear Corporation 416 Hospital, Chengdu, 610051 China

**Keywords:** Esophageal cancer, MUC13, Proliferation, Apoptosis

## Abstract

**Background:**

Esophageal cancer is one of the most common malignant tumors in the world, which is characterized by poor prognosis, aggressiveness, and poor survival. Mucin 13 (MUC13) is a member of the membrane-bound mucin and located on chromosome 3q21.2 and consists of α and β subunits. It has been found that MUC13 is overexpressed in a variety of tumor cells and acts a vital role in the invasiveness and malignant progression of several types of tumors. However, the role and regulatory mechanism of MUC13 in the progression of esophageal cancer remain unclear.

**Methods:**

The expression level of MUC13 was detected in 15 esophageal cancer tissues and 15 pairs of adjacent nontumor tissues by immunohistochemistry (IHC). In addition, the expression of MUC13 mRNA level in human esophageal cancer cell lines (EC9706 and ECA109 and TE-1) was measured by qRT-PCR. In vitro, after silencing MUC13 with lentiviral interference technology, CCK8 assay, clone formation assay, and flow cytometry were applied to investigate the proliferation activity, clone formation ability and anti-apoptosis ability of EC9706 and ECA109 cells. The tumor xenograft growth assay was used to confirm the influence of MUC13 knockdown on the growth of esophageal tumors in vivo. The qRT-PCR assay and western blot experiments were taken to study the mechanism of MUC13 regulating the proproliferation and antiapoptotic of esophageal cancer.

**Results:**

The results showed that MUC13 was overexpressed in esophageal cancer tissues and cell lines (EC9706 and ECA109 and TE-1), especially in EC9706 and ECA109 cells, but low expressed in human esophageal epithelial cell line (HEEC). Next, silencing MUC13 inhibits proliferation, blocks cell cycle progression, and promotes cell apoptosis in vitro, and restrains the growth of esophageal cancer tissues in vivo. Finally, MUC13 affects the proproliferation and antiapoptotic by regulating the expression of GLANT14, MUC3A, MUC1, MUC12, and MUC4 that closely related to O-glycan process.

**Conclusions:**

This study proved that MUC13 is an important molecule that regulates the O-glycan process and then affects the progress of esophageal cancer. MUC13 may be a novel therapeutic target for patients with esophageal cancer.

**Supplementary Information:**

The online version contains supplementary material available at 10.1007/s12672-023-00713-3.

## Background

Esophageal cancer is one of the most malignant cancers, mainly due to its extremely aggressiveness and poor survival rate. Esophageal cancer includes two major malignancies, esophageal squamous cell carcinoma (ESCC) and esophageal adenocarcinoma (EAC). In the examination of esophageal cancer, epithelial dysplasia is considered as a prephase of esophageal squamous cell carcinoma, followed by progression to carcinoma in situ, and eventually to invasive carcinoma [1]. Esophageal adenocarcinoma is usually caused by persistent gastroesophageal reflux, which stimulates intestinal metaplasia of the distal esophageal mucosa [2]. Esophageal squamous cell carcinoma occurs closer to the pharynx (upper 1/3 of the esophagus), while esophageal adenocarcinoma is closer to the junction of the esophagus and stomach below the tracheal bifurcation [2]. There is another difference between esophageal squamous cell carcinoma and adenocarcinoma in disease progression. Squamous cell carcinoma progresses more slowly and has more local recurrence. Adenocarcinoma grows faster and recurrences are more remote and more difficult to control [2, 3]. In recent decades, the application of new technologies, new equipment, and neoadjuvant treatments has made great progress in the prevention and control of esophageal cancer [4, 5]. However, the prognosis of patients suffering from esophageal cancer is poor, and the number of people whose survival time exceeds five years is far from expectations. Therefore, it is urgent to rummage novel therapeutic targets for patients with esophageal cancer.

Mucins are glycoproteins secreted on the surface of cells which can lubricate the epithelial surface of mucosal tissues and provide a chemical barriers. Studies have shown that cell surface mucin 13 ( MUC13) protects the degradation of β-catenin by interacting with GSK-3β, thereby increasing the nuclear translocation of β-catenin and promoting the occurrence, development, invasion and immunosuppression of cancer by signaling transduction [6]. The expression of MUC13 also leads to the activation of NF-κB p65 nuclear translocation and phosphorylation of IκB, which in turn upregulates the expression of important proteins involved in glucose metabolism to promote the invasion of pancreatic cancer [7, 8]. In addition, MUC13 can promote the progression of intrahepatic cholangiocarcinoma through the EGFR/PI3K/AKT pathway [9], distinguish intraductal papillary mucinous tumors from non-mucinous cysts, and is associated with high-risk lesions [10]. Studies have also shown that IL-6 increases the expression of MUC13 by activating the JAK2/STAT5 signaling pathway and promotes the progression of colon cancer [11]. Reducing miR‑132‑3p may promote the spread of gastric cancer by targeting MUC13 [12]. Transcription of MUC13 can enhance the proliferation of glioblastoma stem cells [13]. MUC13 is also abnormally expressed in ovarian cancer and changes the cellular characteristics of SKOV-3 cells [14]. MUC13 is often elevated not only in various malignant tumors but also in certain benign pathologies, so it seems to be non-specific disease biomarker. Nevertheless, MUC13 is significantly elevated in some cancer patients. In this case, its relationship with tumor progression deserves further study [15]. In addition, MUC family proteins are closely related to the process of O-glycans, which exists in a variety of tumors and is of great significance in stimulating tumor cell proliferation and invasion as well as mediating cell adhesion [16–18]. However, the potential function, prognosis, and therapeutic significance of MUC13 in esophageal cancer have not been determined. In this study, we researched the biological and clinical significance of MUC13 in esophageal cancer and its carcinogenic molecular mechanisms, providing a new perspective for esophageal cancer treatment.

## Methods

### Differently expression genes (DEGs) analysis

The cancer public database (Oncomine) was applied to download the original data of sequencing or expression profiling chips and analyzed the DEGs in the cancer tissue and the adjacent nontumor tissue groups. After that, the survival prognostic significance of DEGs in cancer was determined by the Kaplan–Meier plotter, and then Oncomine database was used to confirm the differential expression of DEGs between tumor tissues and normal tissues for the next step in research. In addition, a molecular signaling pathway network regulation map was constructed through using the genes co-expressed with differentially expressed genes in big data, and the related function enrichment map was drawn.

### Cell lines and cell culture

EC9706, TE-1, and ECA109 cell lines used in this experiment were epithelioid cells derived from human esophageal squamous cell carcinoma by primary culture of small tissue blocks. The human esophageal cancer cell lines (EC9706, TE-1, and ECA109) and the human esophageal epithelial cell line (HEEC) were purchased from the Shanghai Cell Bank Collection (Shanghai, China), and cultured in DMEM supplemented with 10% fetal bovine serum (Hangzhou Sijiqing Biological Engineering Materials, China) and maintained in a incubator at 37 °C, 5% CO_2_.

### Immunohistochemistry (IHC)

The paraffin sections of esophageal cancer and adjacent nontumor tissues (HEsoS160CS01, Shanghai Outdo Biotech Co., Ltd.) were deparaffinized with xylene and hydrated with absolute ethanol, 95% ethanol, 80% ethanol, 70% ethanol and distilled water, respectly. After 30 min microwave heat recovery by citric acid antigen retrieval method, the sections were cooled to room temperature and washed with PBS (0.01 M, pH 7.4), and then blocked with 2% BSA blocking solution at room temperature for 2 h. After blocking, the sections were incubated with the primary MUC13 (1: 50, Santa Cruz, USA) antibody for 2 h at 37 °C. Then rinsed three times in PBS, followed by incubation of the secondary antibody (D110087,Sangon Biotech Co., Ltd) at 37 °C for 2 h. DAB substrate kit was used to perform the chromogenic reaction. During which, slices were observed under the microscope to confirm the color end point. Then, the nucleus were counterstained with hematoxylin for 3 min. The slides were dehydrated in a series of graded ethanol solutions and transparented with xylene, and then mounted with a neutral gum. Finally, semiquantitative analysis of immunohistochemical results was conducted by Image ProPlus software, and positive cell percentages were calculated using IHC plug-in after color deconvulutionfor H&E DAB.

### RNA extraction and quantitative real-time PCR

Total RNA was extracted from the cells by using TRIzol^®^ Plus RNA Purification Kit (Invitrogen, USA) and RNase-Free DNase Set (Qiagen, China) in accordance with the manufacturer’s protocol. GAPDH cDNA was used as an internal control for quantification. The primers for qRT-PCR amplification were listed in Table S1.

The conditions of 95 °C for 60 s, 40 cycles of 95 °C for 15 s, 63 °C for 25 s were set for performing PCR.

### Overexpressing and silencing of MUC13

MUC13 cDNA was subcloned into the pCDH-CMV-MCS-EF1-CopGFP-T2A-Puro vector. The MUC13 construction was verified by sequencing. Three small interfering RNA (siRNA) target sequences for MUC13 gene were listed in Table S2 and synthesized by Hippo Biotechnology Co., LTD (Huzhou, China).

Overexpression plasmid of MUC13 transfected into TE-1 cells and three kinds of siRNA were transfected into EC9706 and Eca109 cells. The Overexpressing and silencing efficiency of MUC13 was detected by RT-PCR. The siRNA sequence with the highest knockdown efficiency for MUC13 was cloned into GV115 plasmid vector. The siRNA sequence with the highest knockdown efficiency for MUC13 was 5ʹ- CGACUGUAAGGACAAAUUUCATT-3ʹ (forward) and 5ʹ-UGAAAUUUGUCCUUACAGUCGTT-3ʹ (reverse).

The siRNA constructs were synthesized and cloned into GV115 plasmid vector. The MUC13-siRNA plasmid was then transfected into EC9706 and Eca109 cells with lentivirus following the manufacturer’s protocol.

### Western blot

The proteins were extracted from the cells harvested 72 h after transfection using the total protein extraction kit (Thermo Pierce, USA) and Halt Protease and Phosphatase Inhibitor Cocktail kit (Thermo Pierce, USA), and then the BCA Protein Assay Kit (Biyuntian, Germany) was utilized to measure the consistency of the total proteins. After separating by SDS-PAGE gel, proteins were transferred onto a PVDF membrane (Millipore, USA), which was soaked in methanol for 20 s in advance. The membrane was blocked with 5% BSA at room temperature for 1 h. The primary MUC13, MUC1, MUC4, and GALNT14 antibodies (1:1000, Abcam, UK), GAPDH antibody (1:10000, Abcam, UK), MUC12 antibody (1:500, Santa Cruz, USA) as well as the primary MUC3A antibody (1:200, Santa Cruz, USA) were incubated overnight at 4 °C. After washing with PBS for 5 min, the membrane was incubated at 37 °C for 1 h with a secondary antibody (1:5000, Thermo Pierce, USA). The ECL working solution was prepared following SuperSignal^®^ West Dura Extended Duration Substrate kit instructions and used to develop the transfer film. Finally, the optical density values of the bands were analyzed and the GAPDH was adopted as control.

### CCK8 assay

The amber dehydrogenase in living cells, especially in the mitochondria of proliferating cells, can reduce WST-8 containing in Cell Counting Kit-8 (CCK8) reagent to yellow formazan. The cell proliferation could be evaluated by detecting the absorption value at 450 nm wavelength. In brief, after siRNA transfection of EC9706 and ECA109 cells for 72 h, 100 μL of cell suspension was added and incubated for 24 h at 37 °C. Then, 10 Μl CCK8 was supplemented and incubated for 2 h. At last, the absorbance was gauged at 450 nm on days 1, 2, 3, 4, and 5, respectively.

### Clone formation assay

Clone formation is one of the effective methods for measuring cell proliferation ability. The cells in the logarithmic growth phasewere inoculated in a 6-well plate at a density of 500 cells/well after transfection and continued to culture until the number of cells in most single clones is greater than 50. After the culture were terminated, the cells were fixed with 1 mL of 4% paraformaldehyde for 30 min and stained with 1000 μL of crystal violet staining solution for 20 min. After cells were washed with deionized distilled water (ddH_2_O) for 3 times and images were captured with a digital camera.

### Cell cycle analysis

Cells were harvested with trypsin when they have grown to a coverage rate of about 80%. After centrifuging at 1300 rpm for 5 min, the cells were washed by D-Hanks (pH 7.4) precooled at 4 °C and fixed in 75% ethanol precooled at 4 °C for 2 h. Then, the cells were washed with D-Hanks once again after discarding ethanol. Finally, cells were stained by the working solution (PI: RNase stock solution: D-Hanks = 25: 10: 1000) for 30 min in the dark. The cell solution was collected for cell cycle analysis by flow cytometry (Millipore, USA).

### Apoptosis analysis

Cells were harvested with trypsin after transfection and centrifuged at 300 rpm for 5 min, and resuspended in 200 μL 1 × binding buffer containing 2 μL Annexin V-FITC and 7AAD (PI) for 30 min at room temperature. According to the amount of cells, 200–300 μL 1 × PBS was added to resuspend the cells for apoptosis analysis by flow cytometry (Millipore, USA).

### Xenograft studies

All animal experiments were carried out in strict accordance with the principles and procedures approved by the Laboratory Animal Ethics Committee of Xuzhou Medical University. 4 weeks old female BALB/c nude mice (Shanghai Lingchang Biotechnology Co. Ltd) were selected for xenograft studies. First, 7 × 10^6^/200μL EC9706 cells transfected with siMUC13 or siCtrl were injected subcutaneously into the right hindlimbs of mice. We used the formula: π/6 × (length × width^2^) to calculate tumor size every 2 days and weighed the body weight of the mice (g). At the end of experiment, the mice were euthanized with 2% sodium pentobarbital.

### Statistical analysis

All experiments were repeated three times independently and two-tailed unpaired Student t-test was applied to analyze data. *P* < 0.05 was taken as statistically significant.

## Results

### MUC13 is one hub gene of esophageal cancer

To explore new targets for esophageal cancer, the cancer public database (Oncomine) was applied to confirm the DEGs (Supplementary Figure 1A). It is well known that MUC family proteins are closely related to the process of O-glycans and the occurrence and development of tumors [16–18]. Therefore we selected MUC13 as one hub gene. At present, although there are evidences that MUC13 can be used as biomarkers of esophageal cancer, their functions and mechanisms in depth have not been studied yet [19, 20]. Next, the differential expression of MUC13 between tumor tissues and normal tissues (Supplementary Figure 1B) and the survival prognostic significance of MUC13 (Supplementary Figure 1C) was determined by Oncomine database and the Kaplan–Meier plotter, respectively. After that, it found that MUC13 and its related multiple proteins such as GLANT14, MUC3A, MUC1, MUC12, and MUC4 are closely related to the O-glycan process by PPI protein interaction network analysis (Supplementary Figure 1D) and functional enrichment analysis (Supplementary Figure 1E).

### Overexpression of MUC13 in esophageal cancer tissues and cell lines

**Fig. 1:**
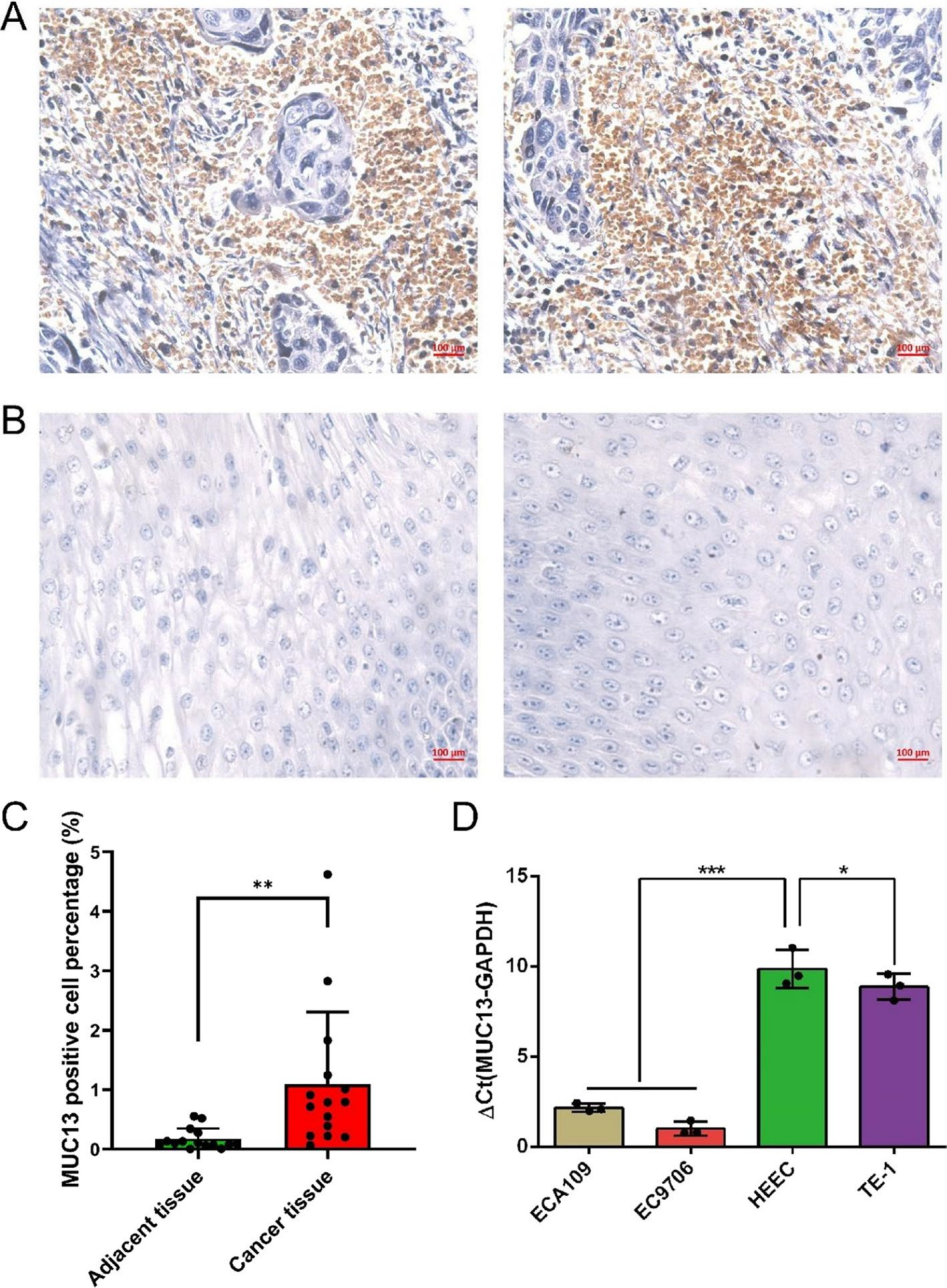
MUC13 expressions in esophageal cancer cell lines. **A** Representative images of immunohistochemical staining for MUC13 in esophageal tumor tissues: strong expression. **B** Representative images of immunohistochemical staining for MUC13 in adjacent non-tumor tissues: absent expression. **C** MUC13 positive cell percentage of Esophageal tumor tissues vs Adjacent non-tumor tissues, n = 30,* P* = 0.01. **D** High expression of MUC13 mRNA level in ECA109 and EC9706 cell lines. Data were expressed as the mean ± s.d., *P < 0.05, ***P < 0.001

The expression level of MUC13 in esophageal cancer tissues and adjacent nontumor specimens was measured by IHC. As is shown in Fig. 1A and B, MUC13 is significantly overexpressed in esophageal tumor samples, but is low expressed in adjacent nontumor tissues. There appears a significant difference in MUC13 positive cell percentage between esophageal cancer tissues and adjacent nontumor specimens (Fig. 1C, n = 30, *P* = 0.01). In vitro experiments, the expression levels of MUC13 in EC9706, TE-1, and Eca109 cell lines were analyzed by qRT-PCR assay. As is shown in Fig. 1D, high expression of MUC13 mRNA level was detected in EC9706, ECA109, and TE-1 cell lines, especially in EC9706 and ECA109 cell lines, but low expression of MUC13 mRNA level was confirmed in TE-1 cell line. We selected EC9706 and ECA109 cell lines to perform subsequent experiments. In all, these findings indicate that MUC13 has overly expression in esophageal cancer.

### Silencing of MUC13 by infection of lentivirus-mediated siRNA in esophageal cancer cells

**Fig. 2:**
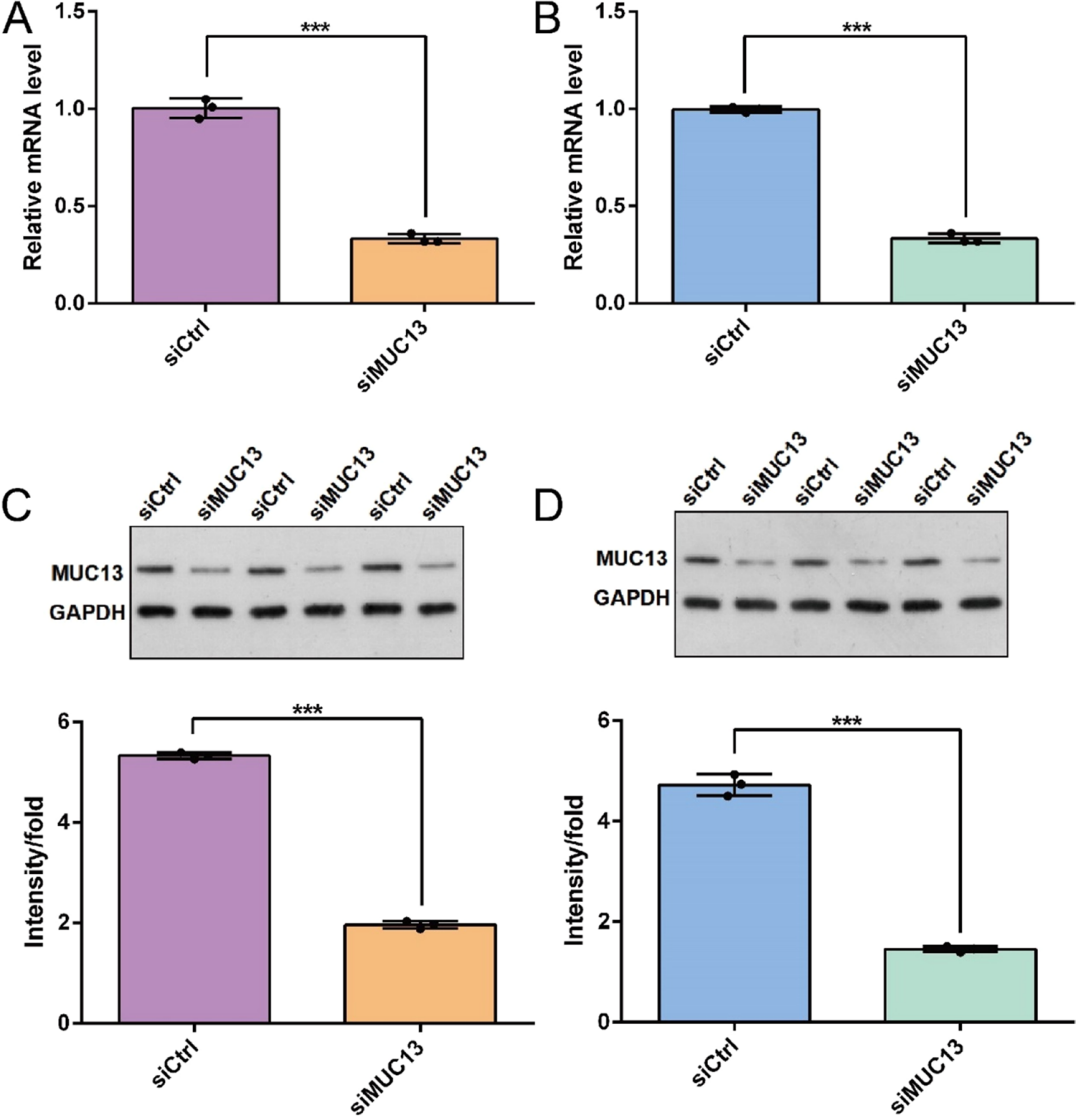
The knockdown efficiency of MUC13 by infection of lentivirus-mediated siRNA and the negative control siRNA in **A** EC9706 cells and **B** ECA109 cells were verified by qRT-PCR. Data were expressed as the mean ± s.d., ***P < 0.001. The knockdown efficiency of MUC13 by infection of lentivirus-mediated siRNA and the negative control siRNA in **C** EC9706 cells and **D** ECA109 cells were verified by Western blot. Data were expressed as the mean ± s.d., ***P < 0.001

MUC13 silence was obtained by transfection with MUC13-specific siRNA plasmids. The qRT-PCR and Western blot were taken to examine the transfection efficiency, respectively. Compared with the negative control siRNA (siCtrl), the mRNA and protein expression levels of MUC13 were both significantly decreased in EC9706 (*P* < 0.001) and ECA109 (*P* < 0.001) cells transfected with MUC13-specific siRNA (siMUC13) (Fig. 2). In brief, silencing of MUC13 was effectively achieved by infection of lentiviral-mediated siRNA in esophageal cancer cells.

### *Overexpressing of MUC13 promoted proliferation and inhibited apoptosis in esophageal cancer cells *in vitro

To comprehensively explore the function of MUC13 gene in esophageal cancer, we overexpressed MUC13 gene in TE-1 cells by transfecting MUC13 overexpression plasmid, and observed the changes of proliferation and apoptosis of TE-1 cells by CCK8 experimental clone formation assay and flow cytometry assay. The qRT-PCR and Western blot were taken to examine the transfection efficiency of the overexpressed plasmid of MUC13. Compared with the negative control plasmid (Ctrl), the mRNA (P < 0.001) and protein (P < 0.01) expression levels of MUC13 in TE-1 were significantly increased (Supplement Figure 2A and B). As shown in Supplementary Figure 2C, the proliferation ability of cells in the MUC13-OE group was significantly enhanced compared with that in the Ctrl group. In addition, overexpression of MUC13 significantly increased (*P* < 0.01) the rate of cell clonal formation (Supplement Figure 2D). Secondly, as shown in Supplement Figure 2E, compared with Ctrl group, the proportion of G1 phase cells in the MUC13-OE group was significantly decreased(*P* < 0.01), while the proportion of cells in S phase and G2/M phase was significantly increased(*P* < 0.05), suggesting that the overexpression of MUC13 promoted the proliferation and division of TE-1 cells. Annexin V-FITC/PI staining by flow cytometry was used to detect the apoptosis rate of cells in Ctrl group and MUC13-OE group. The results showed that the apoptosis ratio of cells in the MUC13-OE group was significantly lower (*P* < 0.001) than that in the Ctrl group (Supplement Figure 2F). In summary, overexpression of MUC13 can promote cell proliferation, clonal formation, and inhibit cell cycle arrest, and apoptosis.

### Silencing of MUC13 inhibited proliferation and promoted apoptosis in esophageal cancer cells in vitro

**Fig. 3:**
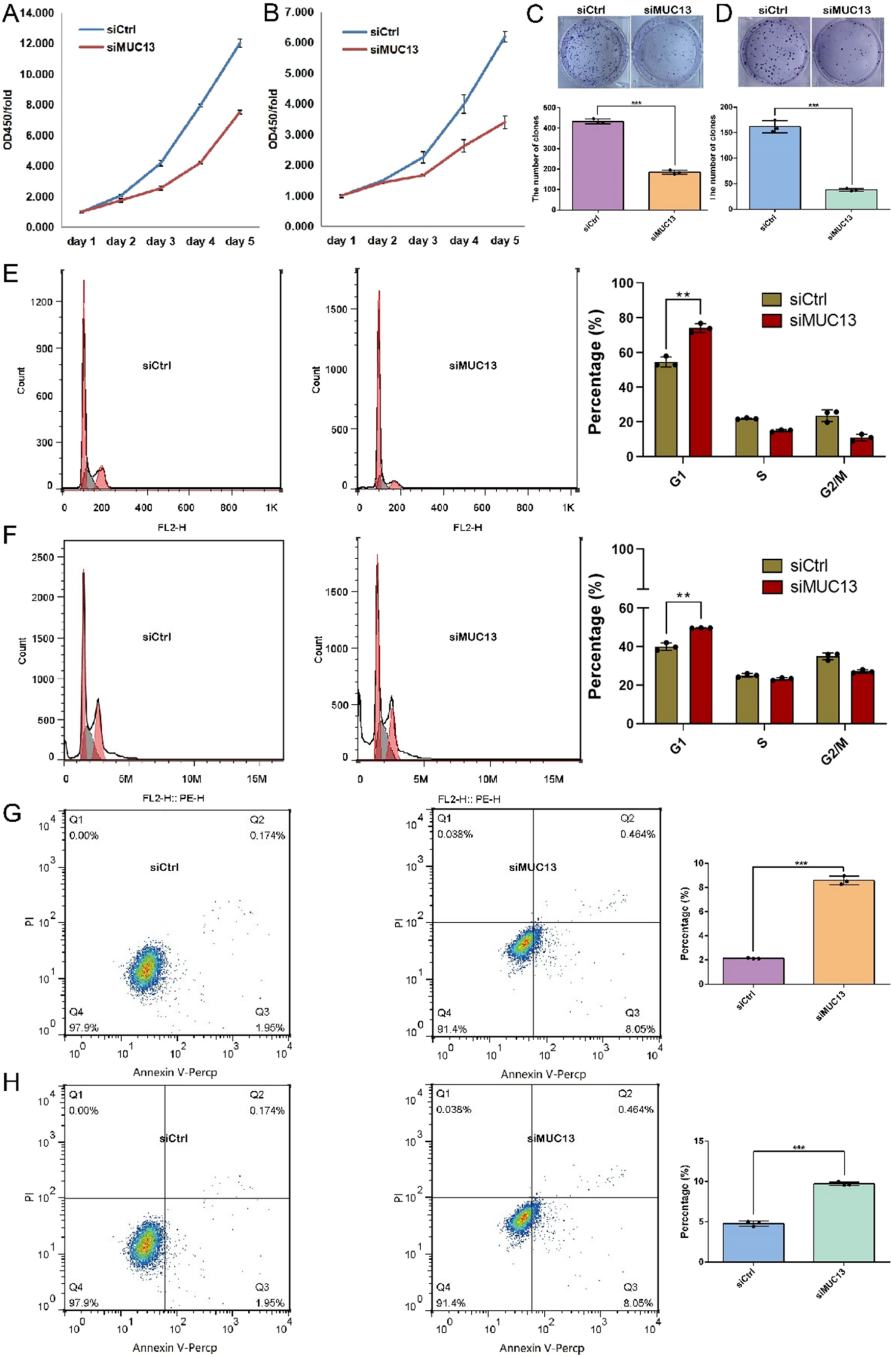
MUC13 knockdown suppressed esophageal cancer cells proliferation and promoted esophageal cancer cells apoptosis. Proliferation of EC907 **A** and ECA109 **B** cells was significantly inhibited in the absence of MUC13. Data were expressed as the mean ± s.d.. The ability of forming colonies of MUC13 knockdown cells was significantly restrained compared with control cells in EC907 **C** and ECA109 **D** cell lines. Data were expressed as the mean ± s.d., ***P < 0.001. The cycle of EC907 **E** and ECA109 **F** cells was significantly arrested in G0/G1 phase in the absence of MUC13. Data were expressed as the mean ± s.d., **P < 0.01. The proportion of apoptotic cells was significantly increased in MUC13 knockdown group compared with control cells in EC907 (**G**) and ECA109 **H** cell lines assessed by flow cytometry. Data were expressed as the mean ± s.d., ***P < 0.001

CCK8 assay, clone formation assay, and cell cycle analysis were used to observe the effects of MUC13 silencing on the proliferation of EC9706 and ECA109 cells. As showed in Fig. 3A and B, compared with siCtrl group, the ability of cell proliferation of siMUC13 group was distinctly declined. Moreover, the cellular clone formation rate of silencing of MUC13 cells was remarkably restrained (*P* < 0.001) in comparison with the siCtrl group (Fig. 3C and D). Next, as illustrated in Fig. 3E and F, compared with the siCtrl group, the proportion of cells in G1 phase was higher in the siMUC13 group, suggesting that the silencing of MUC13 caused the arrest of EC9706(P < 0.01) cells and ECA109(P < 0.01) cells in G1 phase. Finally, the apoptosis rate in siCtrl group or siMUC13 group was detected with Annexin V-FITC/PI staining by flow cytometry. The results showed that the apoptotic proportion was significantly higher in siMUC13 group than in siCtrl group in the EC9706 (*P* < 0.001) and ECA109 (*P* < 0.001) cells (Fig. 3G and H). Overall, it is indicated that silencing of MUC13 could decrease cell proliferation, clone formation, promote cell cycle arrest, and induce cell apoptosis.

### *Silencing of MUC13 suppressed tumor growth *in vivo

**Fig. 4:**
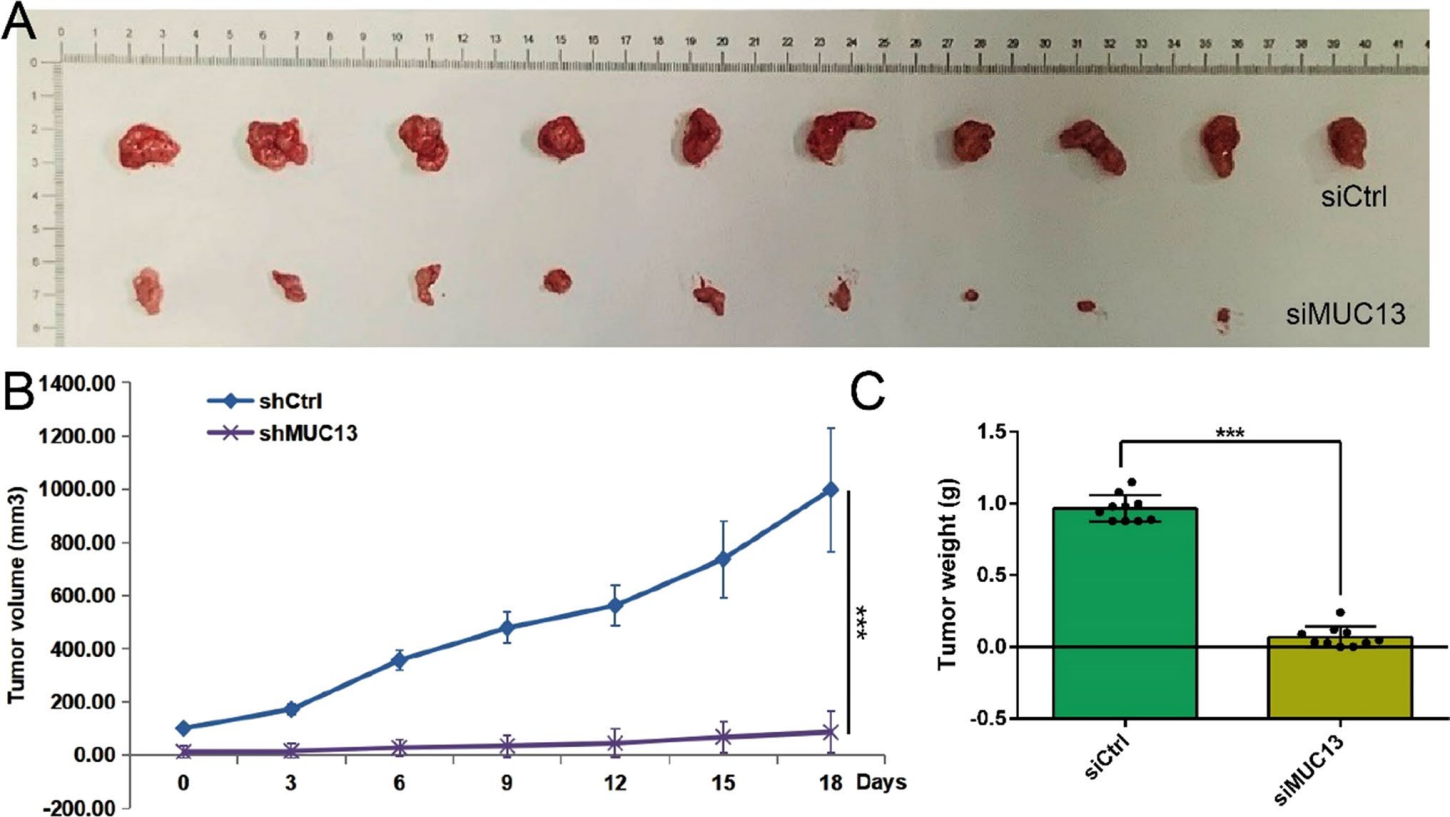
Silencing of MUC13 suppressed tumor growth in vivo. **A** and **B** The tumor volume was obviously decreased in MUC13 knockdown group. Data were expressed as the mean ± s.d., ***P < 0.001. (**C**) The tumor weight was significantly declined in MUC13 knockdown group. Data were expressed as the mean ± s.d., ***P < 0.001

To observe whether silencing of MUC13 reduces the tumorigenicity of esophageal cancer in vivo, EC9706 cells transfected with siMUC13 or siCtrl were subcutaneously inoculated into nude mice. As showed in Fig. 4A, B and C, silencing of MUC13 distinctly decreased the tumor volume and tumor weight, respectly. Therefore, the results proved that MUC13 knockdown restrains the tumorigenicity of esophageal cancer.

### Molecular mechanism analysis

**Fig. 5:**
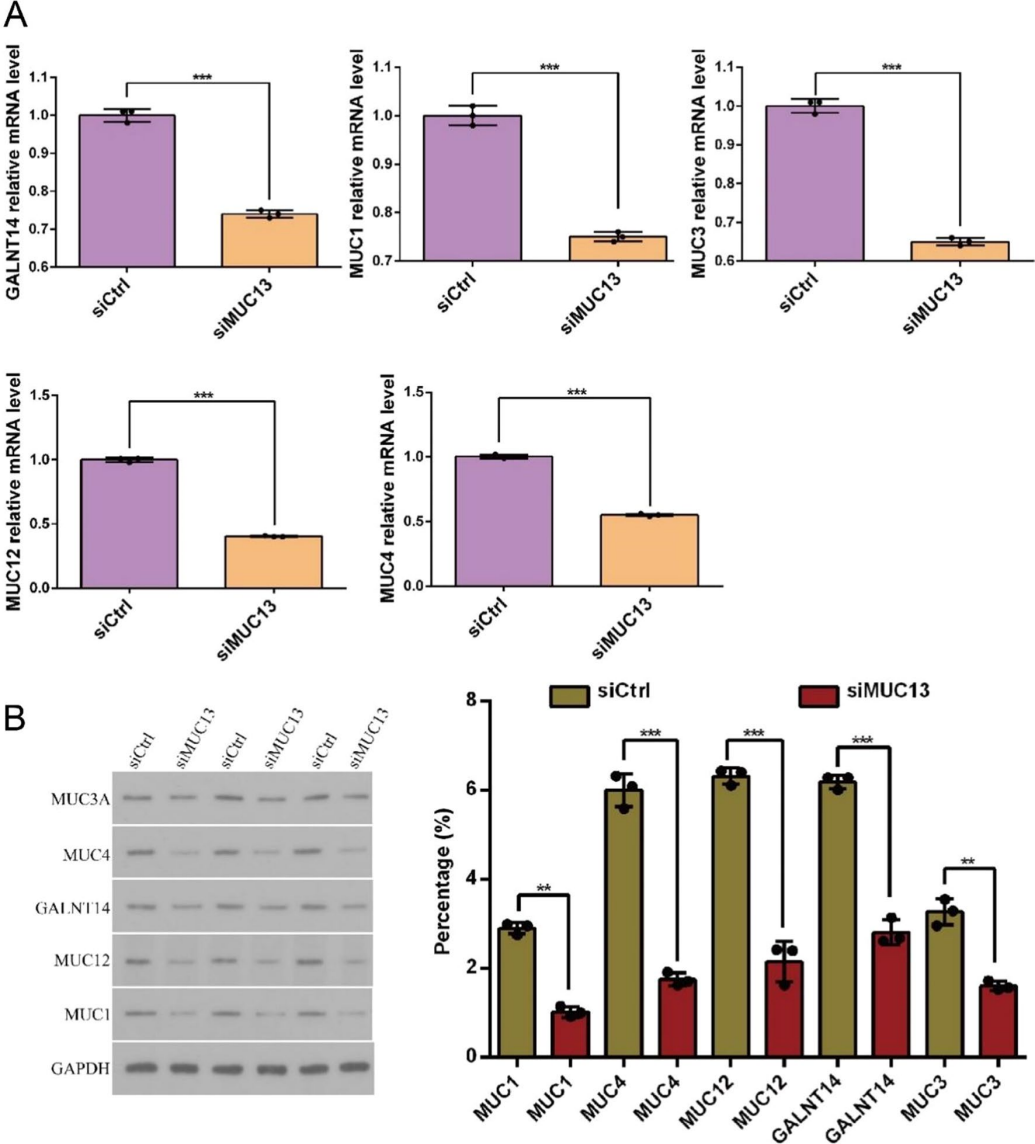
Verification the expression of 5 proteins related to MUC13 in EC9706 cell. **A** GLANT14, MUC3A, MUC1, MUC12 and MUC4 were significantly up-regulated in mRNA level. Data were expressed as the mean ± s.d., ***P < 0.001. **B** GLANT14, MUC3A, MUC1, MUC12 and MUC4 were significantly up-regulated in protein level. Data were expressed as the mean ± s.d., **P < 0.01 and ***P < 0.001

Western blot and qRT-PCR were employed to verify the expression level of 5 proteins (GLANT14, MUC3A, MUC1, MUC12, and MUC4) that related to MUC13 in EC9706 cells. As showed in Fig. 5A and B, compared with siCtrl group, GLANT14, MUC3A, MUC1, MUC12, and MUC4 were significantly downregulated in both protein and mRNA levels in siMUC13 group. In summary, these data indicate correlation between the proproliferation and antiapoptotic effects of MUC13 with the expression of GLANT14, MUC3A, MUC1, MUC12 and MUC4 in esophageal cancer.

## Discussion

The prognosis of esophageal cancer is poor and the incidence has increased in recent years, so it is urgent to find new therapeutic targets for patients with esophageal cancer. Recent research on esophageal cancer has focused on seeking to improve the detection and prediction methods necessary before treatment and emerging multimodality treatment methods [1, 2, 21–23]. Although many studies have demonstrated that MUC13 is abnormally presented in a variety of malignant tumors [6–15], its function and mechanism in the invasiveness and malignant progression of esophageal cancer have not been studied in depth.

As we all know, O-glycan process is closely related to tumor development. Abnormal glycan structures are widely present in a variety of tumors and are of great significance in stimulating tumor cell proliferation and invasion and mediating cell adhesion. It is worth noting that MUC family proteins are closely related to the O-glycan process [16–18]. In this study, we found that the MUC family protein MUC13 gene is the fourth gene based on differential expression level in the public cancer database (Oncomine), but there are few reports on the research of MUC13 in esophageal cancer. In this study, it was found that the MUC family protein MUC13 gene is the fourth gene based on differential expression level in the public cancer database (Oncomine), but there are few reports on the research of MUC13 in esophageal cancer. It also found that MUC13 and its related multiple proteins such as GLANT14, MUC3A, MUC1, MUC12, and MUC4 are closely related to the O-glycan process by PPI protein interaction network analysis and functional enrichment analysis. Therefore we guess that MUC13 can affect the development of esophageal cancer by regulating the O-glycan process, and it is hoped to become a potential therapeutic target of esophageal cancer with certain clinical value.

In this study, we found that MUC13 is highly expressed in some human esophageal cancer tissues compared with adjacent nontumor tissues (Fig. 1A and B). This may be due to the high expression of MUC13, being related to specific types of esophageal cancer, and requires to be further identified. We also found that MUC13 is highly expressed in EC9706 and ECA109 and TE-1 cells and the basal expression level of MUC13 was relatively high, especially in EC9706 and ECA109 cells, while the expression in HEEC cell line was low (Fig. 1C). According to recent studies, MUC13 is overexpressed in gastric cancer cells and liver cancer cells, making it a promising target for cancer therapeutic intervention [12, 24]. Therefore, it is speculated that the characteristics of MUC13 related to cancer can be used for the diagnosis and prognosis prediction of esophageal cancer. However, the relevance between the utterance level of MUC13 and the clinical classification of patients with esophageal cancer needs further study.

Subsequently, we showed that in vitro MUC13 knockdown resulted in a significant range of antiproliferation and pro-apoptosis in EC907 and ECA109 cells. In vivo assays showed that the tumor volume and weight of the siMUC13 group were significantly reduced. These results proved the tumorigenic effect of MUC13 in esophageal cancer. Another study reported that MUC13 knockdown significantly weakened the potential for cell migration and invasion [6, 12], indicating that MUC13 acts as a governing role in maintaining the aggressiveness of cancer. Therefore, the invasion and metastasis of MUC13 in esophageal cancer remain to be further studied.

In previous studies, it was found that MUC13 is abnormally expressed in a variety of malignant tumors [6–15], but its function and mechanism in the invasiveness and malignant progression of esophageal cancer have not been studied in depth. Therefore, it may provide extremely important clues for finding new targets for the treatment of esophageal cancer by studying the underlying mechanism of MUC13 in the development of esophageal cancer. O-glycans are oligosaccharides attached to serine/threonine residues in protein peptide chains and the side chain hydroxyl groups of other amino acid residues. Most of these oligosaccharides are short and present different structures. In this study, we confirmed that GLANT14, MUC3A, MUC1, MUC12, and MUC4 regulatory factors which related to the O-glycan process were overexpressed and downregulated with MUC13 knockdown in esophageal cancer cells, resulting in insufficient tumor growth and proliferation. A large number of studies have also shown that these molecules are involved in the regulation of a variety of malignant tumors, such as MUC4 can be used as a new tumor antigen in pancreatic cancer immunotherapy [28], and MUC12 promotes renal cell carcinoma through the c-Jun/TGF-βsignaling pathway [29], MUC1 confers radioresistance in head and neck squamous cell carcinoma cells [30].But the regulatory network between MUC13 and these molecules needs to be further explored. Abnormal glycan structures are widely present in a variety of tumors and are of great significance in stimulating tumor cell proliferation and invasion and mediating cell adhesion [25–27]. Therefore, it proves that MUC13 as an important molecule regulating the O-glycan process, and could affect the process and development of esophageal cancer in this study. In all, this study lays a foundation for elucidating that MUC13 affects the proliferation and apoptosis of esophageal cancer and indicates MUC13 may be used as a latent therapeutic target for esophageal cancer and has certain clinical value.

Although our study investigated the effect and the potential mechanism of MUC13 on the proliferation and apoptosis of them, the role of MUC13 on the migration and invasion of esophageal cancer cells has not been further studied. Secondly, the expression level of MUC13 has not been detected in a large number of clinical specimens. Therefore, it is necessary to conduct large-scale clinical studies to determine its value in esophageal cancer. Finally, although we have made a preliminary discussion on the mechanism of MUC13 regulation on the proliferation and apoptosis of esophageal cancer cells, further regulation mechanisms have not been studied in depth, and we only selected two strains of esophageal cancer cells for research, which does not have extensive applicability, we need to expand our investigation in the follow-up work.

## Conclusion

**Fig. 6:**
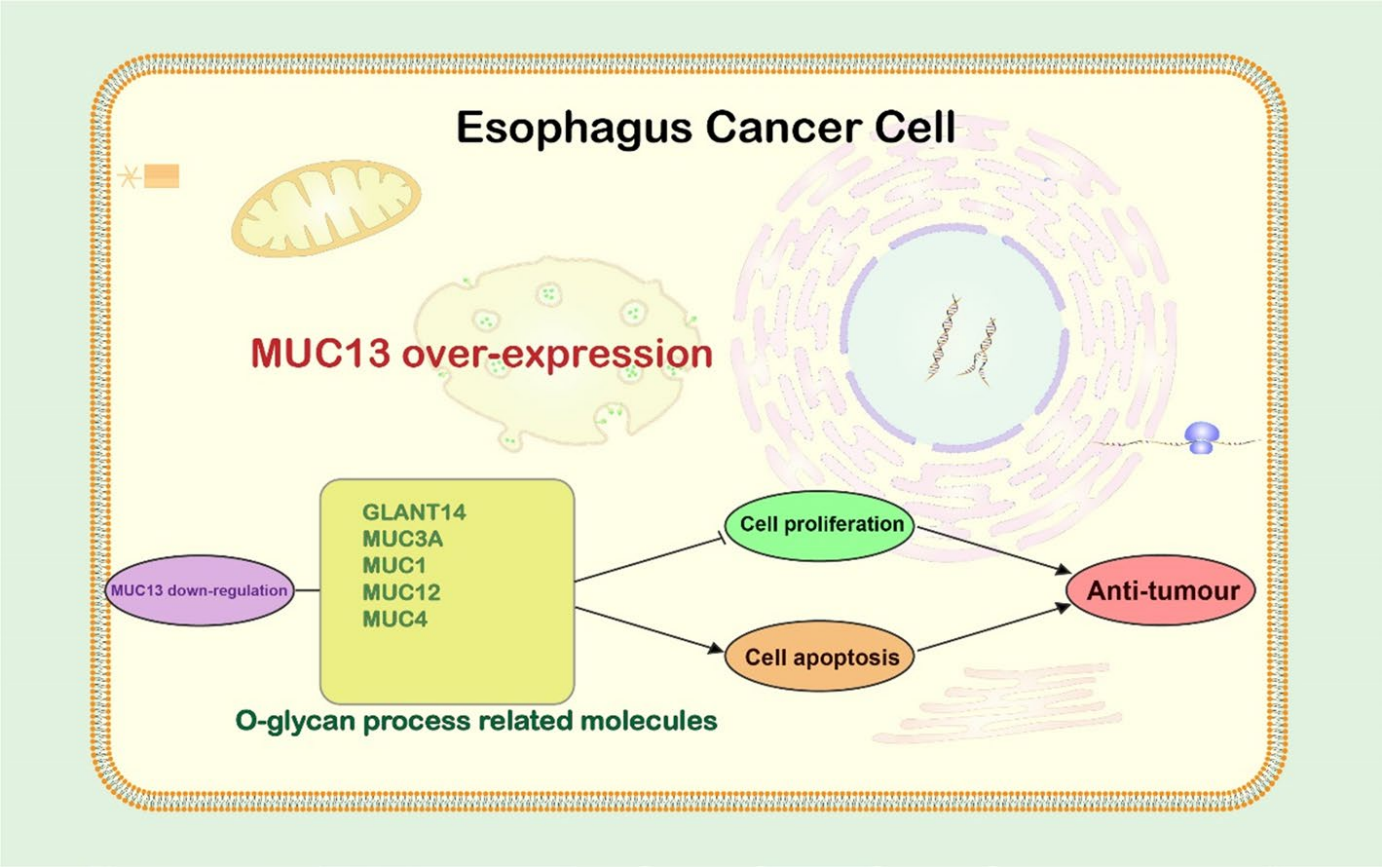
Anti-tumor effect of MUC13 by regulating o-glycan process related molecules

This study proved that MUC13 is an important molecule that regulates the O-glycan process and then affects the progress of esophageal cancer (Fig. 6). MUC13 may be a novel therapeutic target for patients with esophageal cancer.

### Supplementary Information

**Additional file 1**:** Figure S1** MUC13 is one hub gene of esophageal cancer.The differentially expressed genesin the cancer tissue and the adjacent non-tumor tissue group were analyzed by the Oncomine database.The differential expression of MUC13 between tumor tissues and normal tissues was determined by Oncomine database.The survival prognostic significance of MUC13 was determined by the Kaplan-Meier plotter.PPI protein interaction network analysis.Functional enrichment analysis. Figure 2 MUC13 overexpression promoted esophageal cancer cells proliferation and suppressed esophageal cancer cells apoptosis. The overexpression efficiency of MUC13 by infection of MUC13 overexpression plasmid and plasmid vector in TE-1 cells were verified by qRT-PCR. Data were expressed as the mean ± s.d., ***P < 0.001. The overexpression efficiency of MUC13 by infection of MUC13 overexpression plasmid and plasmid vector in TE-1 cells were verified by Western blot. Data were expressed as the mean ± s.d., **P < 0.01. Proliferation of TE-1cells was significantly promoted in the overexpression of MUC13. Data were expressed as the mean ± s.d.. The ability of forming colonies of MUC13 overexpression cells was significantly promoted compared with control cells in TE-1cell lines. Data were expressed as the mean ± s.d., **P < 0.01. The percentage of TE-1cells was significantly reduced in G1 phase in the overexpression of MUC13. Data were expressed as the mean ± s.d., **P < 0.01. The proportion of apoptotic cells was significantly reduced in MUC13 overexpression group compared with control cells in TE-1cell lines assessed by flow cytometry. Data were expressed as the mean ± s.d., ***P < 0.001. TableS1. Primers for qRT-PCR amplification.target sequences for MUC13 gene.DOCX 852 KB)

## Data Availability

The datasets used and/or analysed during the current study are available from the corresponding author on reasonable request.

## Abbreviations

MUC13: Mucin 13
IHC: Immunohistochemistry
HEEC: Human esophageal epithelial cell line
siRNA: Small interfering RNA
CCK8: Cell Counting Kit-8

## Declarations

### ARRIVE guidelines compliance statement

This study is reported in accordance with ARRIVE guidelines.
