# Correction: MUC13 promotes the development of esophageal cancer by upregulating the expression of o-glycan process-related molecules

**DOI:** 10.1007/s12672-023-00771-7

**Published:** 2023-09-11

**Authors:** Yi Han, Gang Chen, Shiyu Liu, Guangqing Zhou, Xinxin Xu, Haihan Zhang, Zhentao Li, Chuannan Wu, Yulan Liu, Kai Fang, Guangxia Chen

**Affiliations:** 1 Department of Gastroenterology, The First People’s Hospital of Xuzhou, Xuzhou Municipal Hospital Affiliated to Xuzhou Medical University, Xuzhou, 221002 China; 2 Department of Plastic Surgery, The Affiliated Hospital of Nanjing University of Chinese Medicine, Nanjing, 210029 China; 3 Xuzhou Medical University, Xuzhou, 221002 China; 4 The Second Affiliated Hospital of Chengdu Medical College, China National Nuclear Corporation 416 Hospital, Chengdu, 610051 China

**Correction: Discover Oncology (2023) 14:123** **https://doi.org/10.1007/s12672-023-00713-3**

The original publication of this article contained several incorrect affiliations. The updated affiliations for the affected authors are shown in this correction article. The original article [1] has been updated.

- Gang Chen, Department of Plastic Surgery, The Affiliated Hospital of Nanjing University of Chinese Medicine, Nanjing, 210029, China
- Guangqing Zhou & Xinxin Xu, Xuzhou Medical University, Xuzhou, 221002, China
- Yulan Liu & Kai Fang, The Second Affiliated Hospital of Chengdu Medical College, China National Nuclear Corporation 416 Hospital, Chengdu, 610051, China

## References

[1] Han Yi, Chen G, Liu S, Zhou G, Xinxin Xu, Zhang H, Li Z, Chuannan Wu, Liu Y, Fang K, Chen G (2023). MUC13 promotes the development of esophageal cancer by upregulating the expression of o-glycan process-related molecules. Discov Oncol.

