# Supplementary material for: MUC13 promotes the development of esophageal cancer by upregulating the expression of o-glycan process-related molecules
Source: Discov Oncol. 2023 Jul 3;14:123. doi: 10.1007/s12672-023-00713-3 (PMC10317945; doi:10.1007/s12672-023-00713-3)
Supplement: Supplementary file 1 — Additional file 1: Figure S1 MUC13 is one hub gene of esophageal cancer.The differentially expressed genesin the cancer tissue and the adjacent non-tumor tissue group were analyzed by the Oncomine database.The differential expression of MUC13 between tumor tissues and normal tissues was determined by Oncomine database.The survival prognostic significance of MUC13 was determined by the Kaplan-Meier plotter.PPI protein interaction network analysis.Functional enrichment analysis. Figure 2 MUC13 overexpression promoted esophageal cancer cells proliferation and suppressed esophageal cancer cells apoptosis. The overexpression efficiency of MUC13 by infection of MUC13 overexpression plasmid and plasmid vector in TE-1 cells were verified by qRT-PCR. Data were expressed as the mean ± s.d., ***P < 0.001. The overexpression efficiency of MUC13 by infection of MUC13 overexpression plasmid and plasmid vector in TE-1 cells were verified by Western blot. Data were expressed as the mean ± s.d., **P < 0.01. Proliferation of TE-1cells was significantly promoted in the overexpression of MUC13. Data were expressed as the mean ± s.d.. The ability of forming colonies of MUC13 overexpression cells was significantly promoted compared with control cells in TE-1cell lines. Data were expressed as the mean ± s.d., **P < 0.01. The percentage of TE-1cells was significantly reduced in G1 phase in the overexpression of MUC13. Data were expressed as the mean ± s.d., **P < 0.01. The proportion of apoptotic cells was significantly reduced in MUC13 overexpression group compared with control cells in TE-1cell lines assessed by flow cytometry. Data were expressed as the mean ± s.d., ***P < 0.001. TableS1. Primers for qRT-PCR amplification.target sequences for MUC13 gene.DOCX 852 KB) [file 12672_2023_713_MOESM1_ESM.docx]

**Supplementary Figure 1** MUC13 is one hub gene of esophageal cancer. (**A**) The differentially expressed genes (DEGs) in the cancer tissue and the adjacent non-tumor tissue group were analyzed by the Oncomine database. (**B**) The differential expression of MUC13 between tumor tissues and normal tissues was determined by Oncomine database. (**C**) The survival prognostic significance of MUC13 was determined by the Kaplan-Meier plotter. (**D**) PPI protein interaction network analysis. (**E**) Functional enrichment analysis.


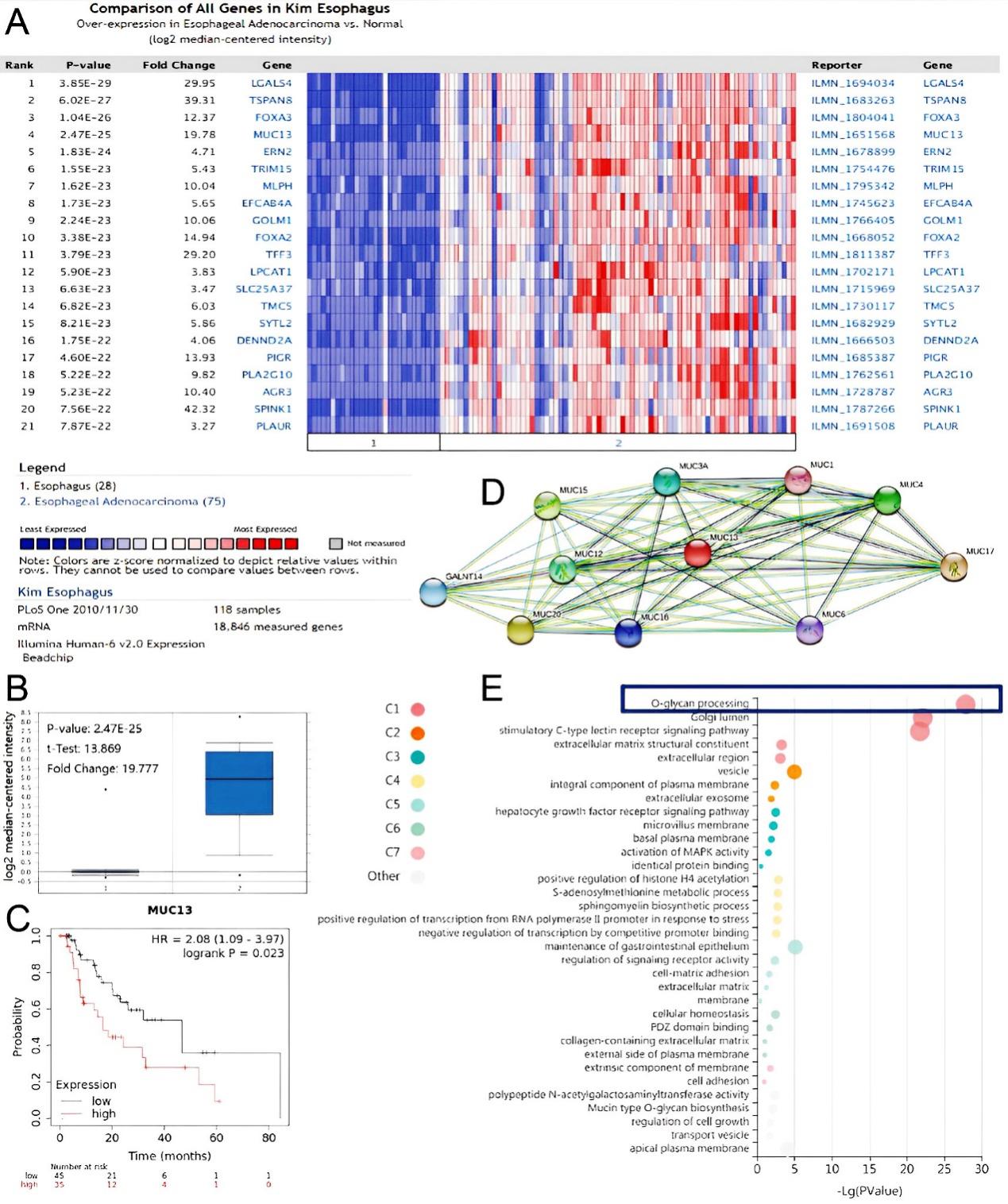


**Supplementary Figure 2** MUC13 overexpression promoted esophageal cancer cells proliferation and suppressed esophageal cancer cells apoptosis. The overexpression efficiency of MUC13 by infection of MUC13 overexpression plasmid and plasmid vector in TE-1 cells were verified by qRT-PCR (A). Data were expressed as the mean ± s.d., ****P* < 0.001. The overexpression efficiency of MUC13 by infection of MUC13 overexpression plasmid and plasmid vector in TE-1 cells were verified by Western blot (B). Data were expressed as the mean ± s.d., ***P* < 0.01. Proliferation of TE-1 (C) cells was significantly promoted in the overexpression of MUC13. Data were expressed as the mean ± s.d.. The ability of forming colonies of MUC13 overexpression cells was significantly promoted compared with control cells in TE-1 (D) cell lines. Data were expressed as the mean ± s.d., ***P* < 0.01. The percentage of TE-1 (E) cells was significantly reduced in G1 phase in the overexpression of MUC13. Data were expressed as the mean ± s.d., ***P* < 0.01. The proportion of apoptotic cells was significantly reduced in MUC13 overexpression group compared with control cells in TE-1 (F) cell lines assessed by flow cytometry. Data were expressed as the mean ± s.d., ****P* < 0.001.


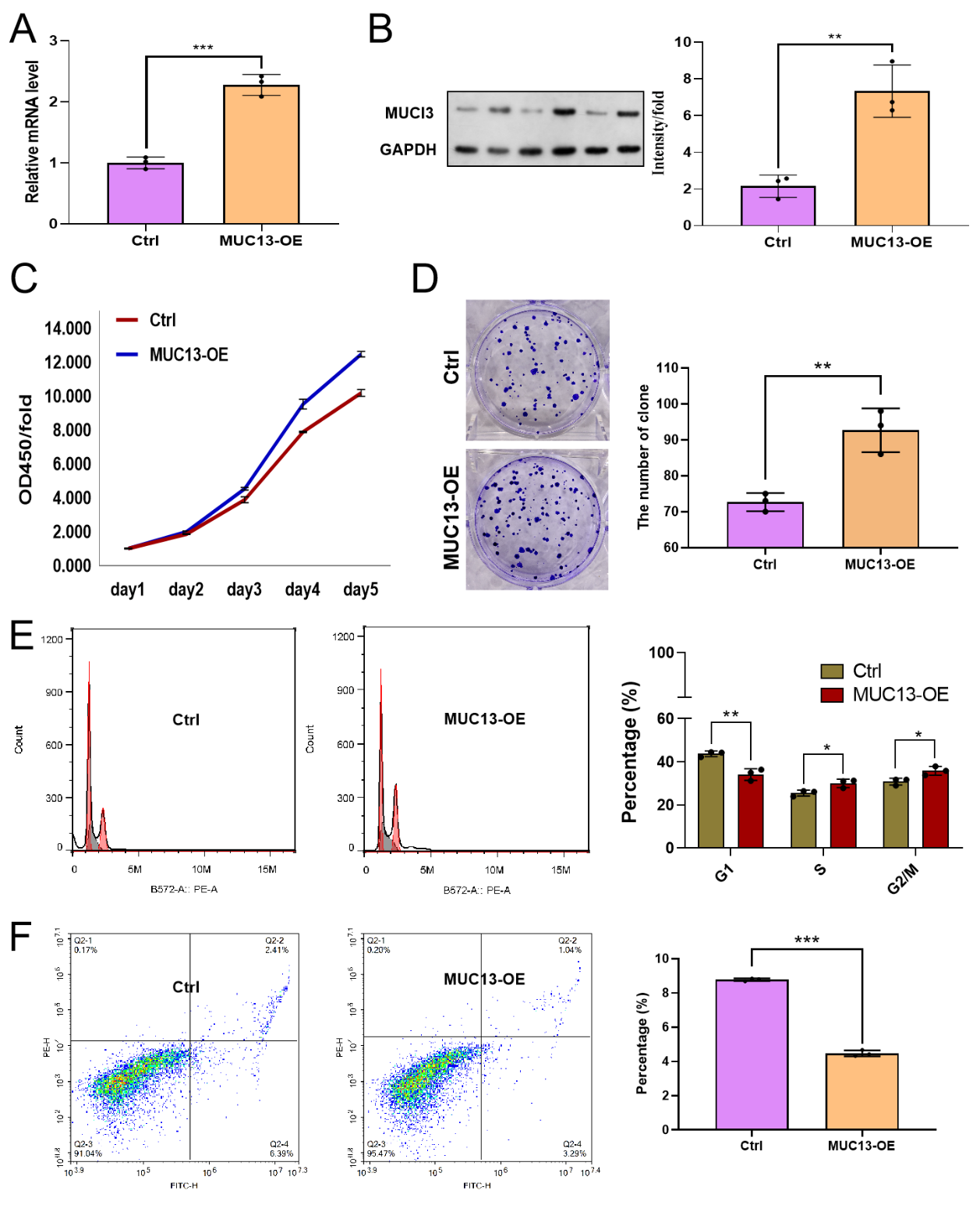


| **TableS1. Primers for qRT-PCR amplification** | | | |
| --- | --- | --- | --- |
| Gene name | ID | Forward (5’-3’) | Reverse (5’-3’) |
| MUC13 | NM_033049.4 | TCAAGTGTCCTGATGCCTGC | GCTCCCTTCTGCTCCAAGAT |
| GAPDH | BC059110 | AGAAGGCTGGGGCTCATTTG | AGGGGCCATCCACAGTCTTC |
| MUC1 | NM_001018016.3 | TGCCGCCGAAAGAACTAC | TGCCGCCGAAAGAACTAC |
| MUC4 | NM_001322468.1 | CAGGGACGACGGGACTTA | ACAGGGCACAGAGGTAGGG |
| MUC12 | NM_001164462.2 | GAACGAAGTCGCAAATGA | TGGGATAGGCTGAATAAGAT |
| MUC3A | NM_005960.2 | TTTCGAGGACGACGGAACAG | TCACACTGAGGACGAGGTCA |
| GLANT14 | NM_001253826.2 | CCCCAGTGGTTCTTGTCC | GGATGTCTGAGGTGGTTGC |

| **TableS2. Small interfering RNA (siRNA) target sequences for MUC13 gene** | | |
| --- | --- | --- |
| Name | Forward (5’-3’) | Reverse (5’-3’) |
| MUC13 siRNA-1 | GGCAACUCAGCUGAUGCUGUATT | UACAGCAUCAGCUGAGUUGCCTT |
| MUC13 siRNA-2 | CCUGUGCAGAUAAUUCGUUAUTT | AUAACGAAUUAUCUGCACAGGTT |
| MUC13 siRNA-3 | CGACUGUAAGGACAAAUUUCATT | UGAAAUUUGUCCUUACAGUCGTT |
